# Supplementary material for: Association of Human FOS Promoter Variants with the Occurrence of Knee-Osteoarthritis in a Case Control Association Study
Source: Int J Mol Sci. 2019 Mar 19;20(6):1382. doi: 10.3390/ijms20061382 (PMC6471183; doi:10.3390/ijms20061382)
Supplement: Supplementary file 1 [file ijms-20-01382-s001.zip › ijms-447789-SI.pdf]

Supplementary Materials

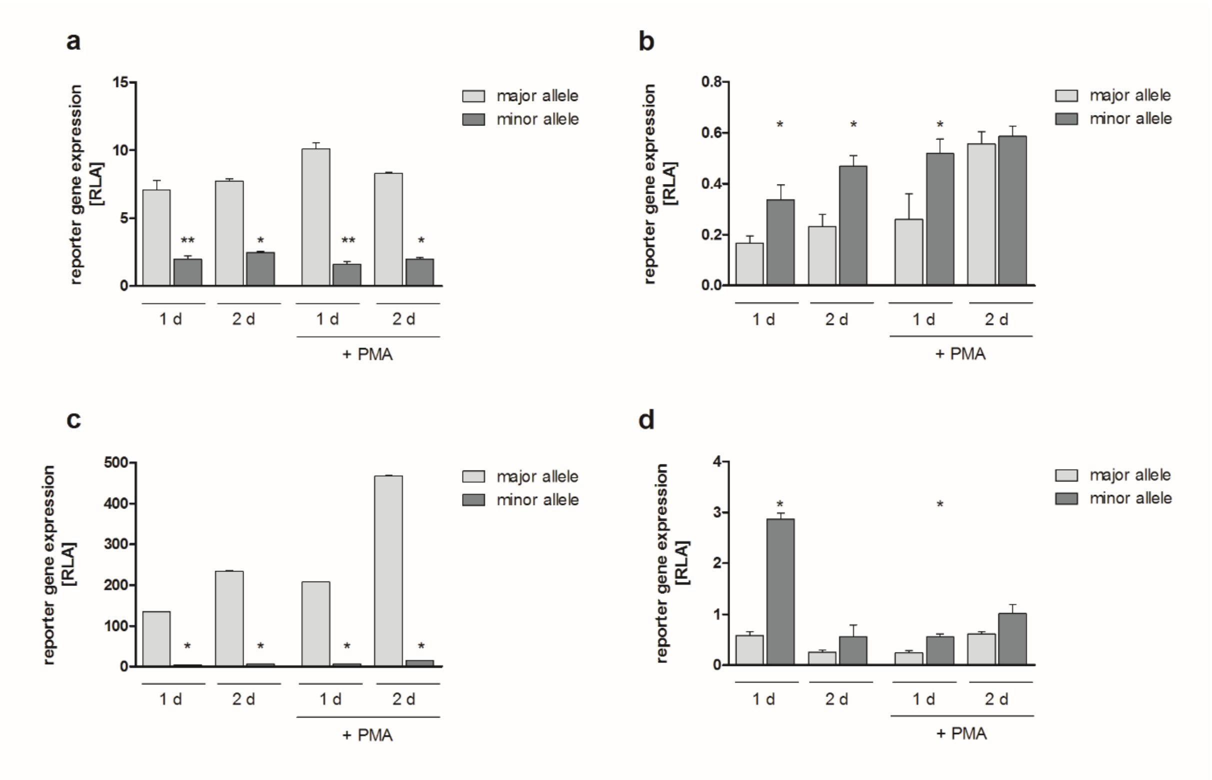

Figure S1. Reporter gene expression in K4IM and HeLa cells. The graph shows absolute levels (in RLA) of *JUN* (**a, c**) and *FOS* (**b, d**) promoter-dependent expression of firefly luciferase 1d and 2d following transfection of K4IM human fibroblasts (**a, b**) or HeLa human epithelial cells (**c, d**), either in non-stimulated cells or in cells stimulated with 10 ng/ml PMA for 8 h (determined in biological triplicates each; in d, 48 h values: duplicates; mean ± SEM). Firefly luciferase expression levels were normalized to renilla luciferase expression levels in the respective samples (transfection and normalization control). ** $P \leq 0.005$, * $P \leq 0.05$ compared to the major allele. For *JUN*, the major allele is the rs4647009-deletion, for *FOS*, the major allele refers to the combination of rs7101-T and rs2239615-T.

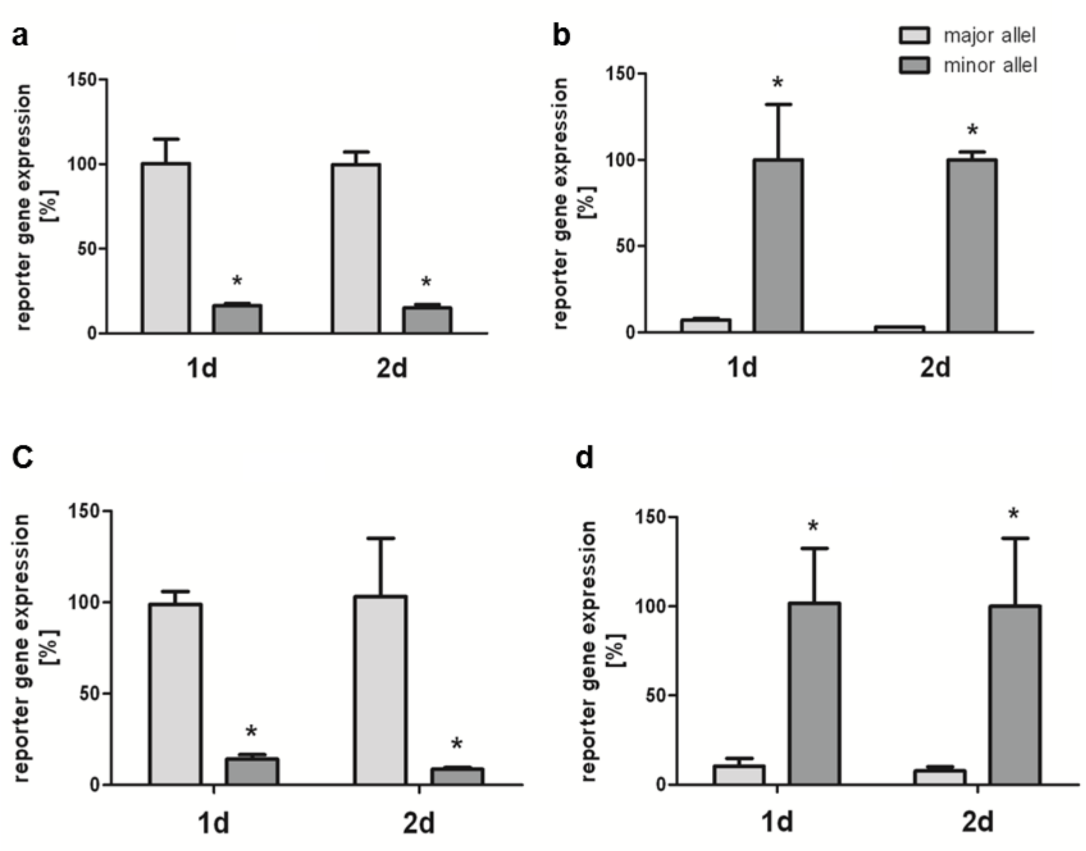

Figure S2. Reporter gene expression in NIH3T3 cells. The graph shows *JUN* (**a, c**) and *FOS* (**b, d**) promoter-dependent expression of firefly luciferase 1d and 2d following transfection of NIH-3T3 murine embryonic fibroblasts, either (**a, b**) in non-stimulated cells or (**c, d**) in cells stimulated with 10 ng/ml PMA for 8h (determined in biological triplicates each). Firefly luciferase expression levels were normalized to renilla luciferase expression levels in the respective samples (transfection and normalization control). Expression levels are shown as relative values (in %). Error bars represent standard errors. * $P \leq 0.05$ compared to the major allele.

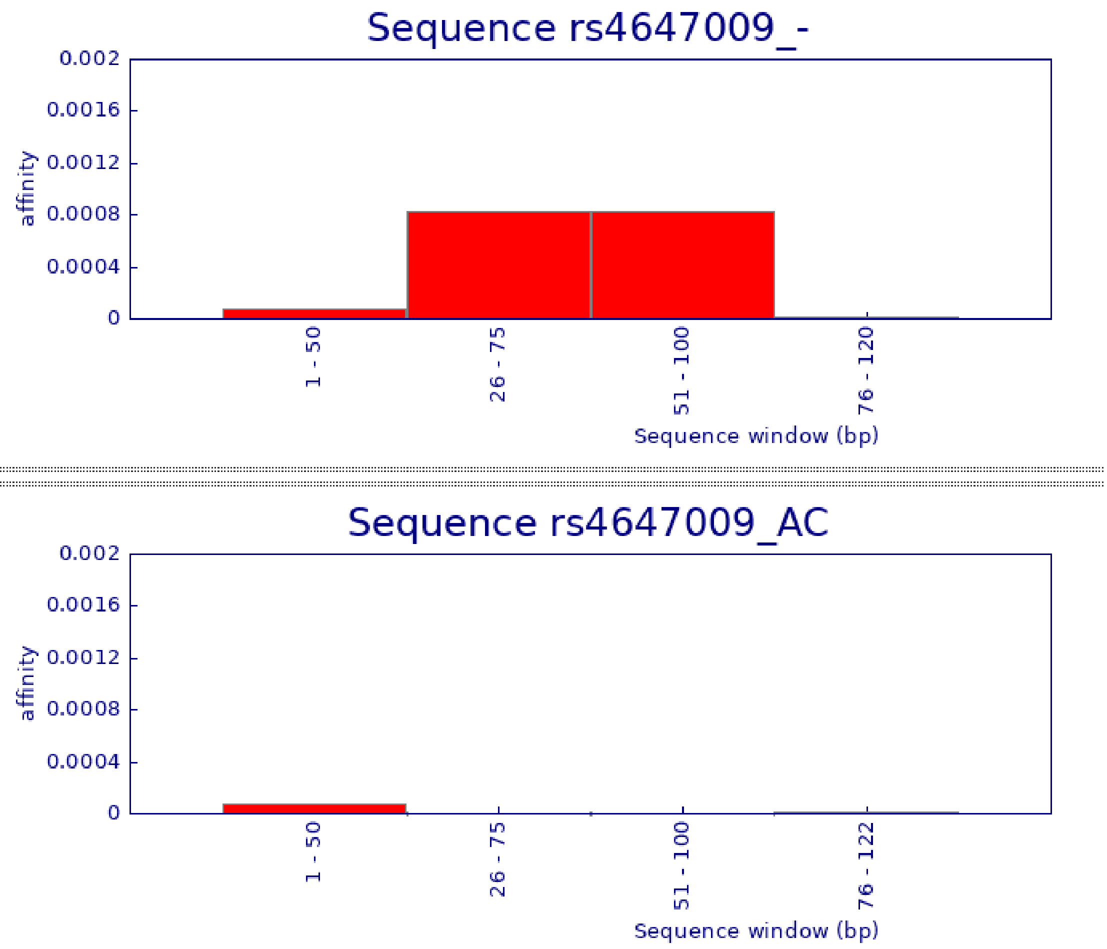

Figure S3. Affinity plot showing differential allelic modulation of transcription factor NF-κB binding site (Model ID: M00208) for variant rs4647009. Shown are the results for the major allele (i.e., the deletion „-“; upper plot, *P*=0.02) and for the minor allele (i.e., the insertion „AC“; lower plot, *P*=0.09). Analysis was done using the tool sTRAP with standard-settings (PMID: 20127973; available at http://trap.molgen.mpg.de/cgi-bin/trap_two_seq_form.cgi).

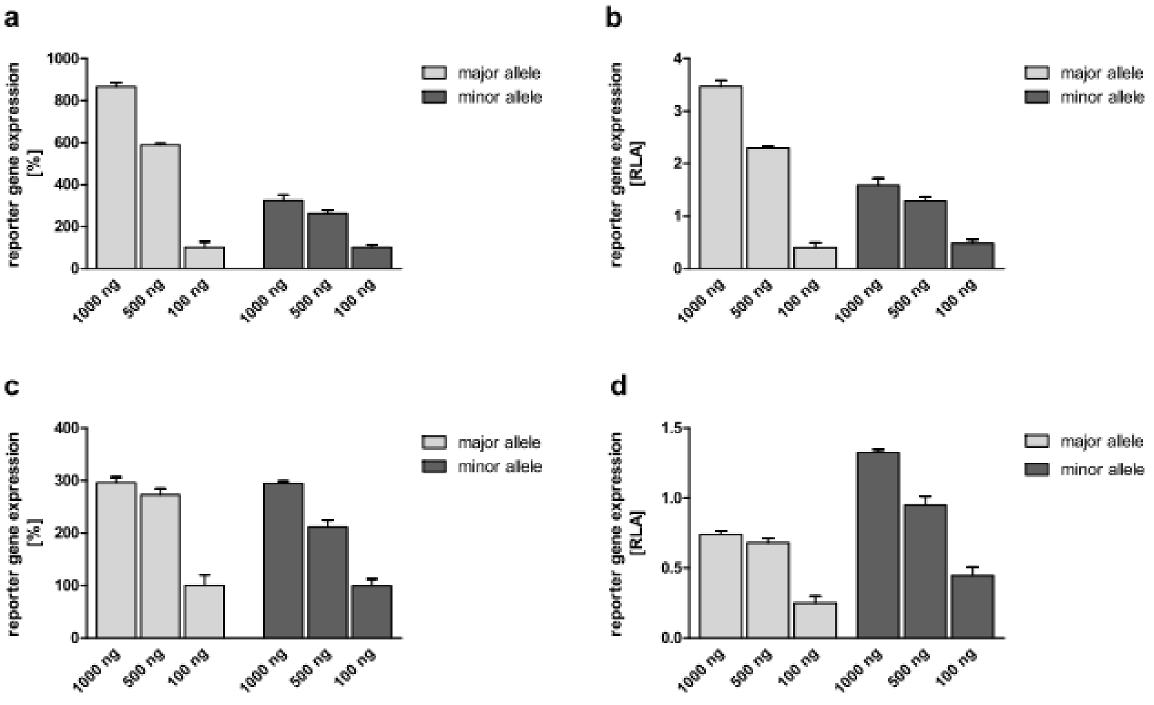

Figure S4. Dose response experiments. The graph shows *JUN* (**a**, **b**) and *FOS* (**c**, **d**) promoter-dependent expression of firefly luciferase 1d after transfection of K4IM human fibroblasts with 1000, 500, or 100 ng vector per well (determined in biological triplicates each; mean ± SEM). Firefly luciferase expression levels were normalized to renilla luciferase expression levels. Expression levels are shown as relative values (in %; **a**, **c**) and absolute values (in RLA; **b**, **d**).

Table S1. Validated SNPs in promoter regions (dbSNP build 130)

Gene	rs identifyer	Allelic frequency	Analysed chromosomes	Population	db SNP validation status	Identified in initial screening population
FOS	rs2239615 **(FOS -135)**	0.30	400	Caucasian	by-cluster,by-frequency	**yes**
FOS	rs7101 **(FOS -60)**	0.26	400	Caucasian	by-cluster,by-frequency,by-submitter,by-2hit-2allele,by-hapmap	**yes**
FOS	rs4645850	0.07	120	Caucasian	by-cluster,by-frequency	no
FOS	rs2234706	0.03	400	Caucasian	by-cluster,by-frequency	no
FOS	rs4645849	0.02	168	Global	by-frequency	no
FOS	rs4645852	0.01	138	Global	by-frequency	no
JUN	**rs4647001 (JUN -1676)**	0.39	156	Global	by-cluster,by-frequency,by-2hit-2allele	**yes**
JUN	rs4646999	0.33	48	Caucasian	by-cluster,by-frequency,by-2hit-2allele	no
JUN	rs4647002	0.05	172	Global	by-cluster,by-frequency	no
JUN	rs4647000	0.04	168	Global	by-cluster,by-frequency	no
JUN	rs4647009 **(JUN -617/-618)**	0.03	62	Caucasian	by-cluster,by-frequency	**yes**
JUN	rs2760499	0.03	162	Global	by-cluster,by-frequency	no
JUN	rs4647011	0.02	156	Global	by-frequency	no
JUN	rs4647003	0.01	166	Global	by-frequency	no
JUNB	rs17886698	0.04	170	Global	by-frequency	no
JUNB	rs17881432	0.04	156	Global	by-frequency	no
JUNB	rs17878468	0.03	166	Global	by-frequency	no

Gene	rs identifyer	Allelic frequency	Analysed chromosomes	Populatio n	db SNP validation status	Identified in initial screening population
JUNB	rs17883538	0.02	170	Global	by-frequency	no
JUN D	rs41523455	0.39	44	Caucasian	by-frequency	no
JUN D	rs7247222	0.36	44	Caucasian	by-cluster,by-frequency	no
JUN D	rs7247237	0.25	44	Caucasian	by-cluster,by-frequency	no
JUN D	rs7247767	0.25	44	Caucasian	by-cluster,by-frequency	no
JUN D	rs41507248	0.25	44	Caucasian	by-frequency	no
JUN D	rs41519246	0.21	44	Caucasian	by-frequency	no
JUN D	rs41374745	0.18	44	Caucasian	by-frequency	no
JUN D	rs6512255	0.17	120	Caucasian	by-cluster,by-frequency,by-2hit-2allele,by-hapmap	no

Bold SNPs were identified in the initial screening study (50 chromosomes) consisting of RA, OA, and NC samples.

Table S2. Clinical characteristics of the donors

		normal controls (NC)	osteoarthritis (OA)	rheumatoid arthritis (RA)
donors (n)		484	277	298
gender (female/male)		226/258	204/84	216/82
age (years ± SEM)		41.2 ± 0.5	69.1 ± 0.5	59.7 ± 0.7
disease duration (years ± SEM)		-	5.9 ± 0.4 (n.d.: 5)	11.5 ± 0.6 (n.d.: 2)
rheumatoid factor (positive/negative/n.d.)		0/0/484	21/267/0	219/54/25
ESR[1] (mm/h ± SEM)		n.d.	16.4 ± 0.8	26.4 ± 1.2 (n.d.: 4)
CRP[2] (mg/l ± SEM)		n.d.	7.6 ± 1.3	17.3 ± 1.4 (n.d.: 17)
ARA[3] - criteria for RA (n ± SEM)		n.d.	0.1 ± 0.01	4.7 ± 0.1 (n.d.: 2)
Concomitant medication[4]	MTX[5] (n)	-	0	193
	Steroids	-	[illegible]2	183
	NSAIDs[6] (n)	-	141	230

n.d. not determined

[1] Erythrocyte sedimentation rate

[2] C-reactive protein, normal range: < 5 mg/l

[3] American Rheumatism Association (now: American College of Rheumatology)

[4] n.d.: 8 (RA)

[5] Methotrexate

[6] non-steroidal anti-inflammatory drugs

Table S3. Clinical characteristics of the replication cohort [1].

		normal controls (NC)	osteoarthritis (OA)
donors (n)		548	72
gender (female/male)		276/272	48/24
age (years ± SD)		58.9 ± 6.8	62.3 ± 7.5
disease duration (years ± SD)			7.9 ± 9.1
rheumatoid factor (positive/negative/n.d.)		0 / 548 / 0	0 / 72 / 0
CRP[2] (mg/l ± SD)		2.1 ± 5.3	3.2 ± 3.6
Rheumatoid arthritis[3]		0	0
Concomitant medication[4]	MTX[5] (n)	0	0
	Beta-agonist and corticosteroid combination	0	0
	Systemic corticosteroids	0	0
	Inhaled corticosteroids	0	7
	NSAID[6]	0	42

1 Additional information regarding selection of individuals from the "Genmets" study can be found in section Material and Methods
2 C-reactive protein, normal range: < 5 mg/l
3 Diagnostic details as described in [44]
4 Regular or irregular use at the time of investigation for any reason
5 Methotrexate
6 non steroidal anti-inflammatory drugs, includes prescribed and over-the-counter

Table 4. Association analysis of rs2239615/rs7101 in *FOS* with knee-OA in the replication cohort without excluding individuals using NSAIDs, systemic/inhaled corticosteroids, and/or having elevated levels of rheumatoid factor (RF >30 IU/ml) (in total 893 NC and 75 knee-OA individuals).

	NC cohort	OA cohort
Homozygous minor (C/C)	31	4
Heterozygous (C/T)	279	35
Homozygous major (T/T)	583	36
HWE *P*-value	0.82	0.27
allelic OR (95% CI)		1.70 (1.2-2.5)
allelic OR - *P*-value		0.0074
Minor Recessive OR (95% CI)		1.57 (0.5-4.6)
Minor Recessive OR *P*-value		0.34

Supplement

Table S5. primer sequences and specific PCR conditions for NIRCA analyses (cloning into vector pUC 19). primer forward: binding site for T7 RNA polymerase + *Eco* RI restriction site + sequence for primer binding; primer reverse: binding site for T7 RNA polymerase + *Bam* HI restriction + sequence for primer binding.

**promoter**	**primer forward** (5′→3′)	**primer reverse** (3′→5′)	**product** (bp)	**amplification protocol** (45 cycles)
JUN promoter I	5′- AGAGCCTGGTCTCCAGCCGCCCC - 3′ (position: -779)	5′- TGCCCGTTGCTGGACTGGATTATC -3′ (position: +260)	1039	denaturation: 45 s, 95°C, primer annealing: 45 s, 60°C, amplification: 180 s, 72°C
JUN promoter II	5′- ACCGTCGCTCCTGAA -3′ (position: -1736)	5′- GCCACTTGTCTCCGGGT -3′ (position: -619)	1117	denaturation: 45 s, 95°C, primer annealing: 45 s, 55°C, amplification: 180 s, 72°C
JUNB promoter I	5′- TCCTCCGTCCTGTGAAAATTCCAG -3′ (position: -842)	5′- CGCTTTGAGACTCCGGTAGGGGTC -3′ (position: +156)	1050	denaturation: 45 s, 95°C, primer annealing: 45 s, 53°C, amplification: 180 s, 72°C
JUNB promoter II	5′- CCTGTGCCCTAATATGGGCGGC -3′ (position: -1831)	5′- TCCCAGTATGTGCGAAGAAACC -3′ (position: -640)	1192	denaturation: 45 s, 95°C, primer annealing: 45 s, 56°C, amplification: 180 s, 72°C

**promoter**	**primer forward** (5′→3′)	**primer reverse** (3′→5′)	**product** (bp)	**amplification protocol** (45 cycles)
JUND promoter I	5′- AGATCGGTCGTACACAGCGGT -3′ (position: -400)	5′- CAGCGTCAGCGCGTCCTTCTTCATC -3′ (position: +156)	556	Denaturation: 45 s, 95°C, primer annealing: 45 s, 59°C, amplification: 180 s, 72°C
JUND promoter II	5′- CCATTCTATGCGAGGCCCTGTCA -3′ (position: -1629)	5′- GCGTGATGGGCCCGGGCCAC -3′ (position: -438)	1192	denaturation: 45 s, 95°C, primer annealing: 45 s, 57,8°C, amplification: 180 s, 72°C
FOS promoter I	5′ - CATATTAGGACATCTGCGTC - 3′ (position: -468)	5′- CTGCGCGTTGACAGGCGAGCC -3′ (position: +141)	609	denaturation: 45 s, 95°C, primer annealing: 45 s, 56°C, amplification: 90 s, 72°C
FOS promoter II	5′ - CACCCCCTTCAAATGTCTTC - 3′ (position: -1109)	5′- GGTTTCGGGGATGGCT -3′ (position: -395)	715	denaturation: 45 s, 95°C, primer annealing: 45 s, 45,5°C, amplification: 90 s, 72°C
FOS promoter III	5′ - AGCCCCGTGTTCCAGGACGTG - 3′ (position: -1410)	5′- AGACCTTCATCCCCTAACCTC -3′ (position: -830)	581	denaturation: 45 s, 95°C, primer annealing: 45 s, 60°C, amplification : 90 s, 72°C
FOS promoter IV	5′ - GTTCCACGAATCCCCGCCTC - 3′ (position: -2008)	5′- AGGGTGGAGGACGGGGGCTG -3′ (position: -1305)	704	denaturation: 45 s, 95°C, primer annealing: 45 s, 65°C, amplification: 90 s, 72°C

bp: base pairs; position +1: first base of the start codon **A**TG

Table S6. primer sequences and specific PCR conditions for functional analyses (cloning into vector pUBT-luc).

primer forward: *Hind* III restriction site + sequence for primer binding

primer reverse: *Not* I restriction + sequence for primer binding

**promoter**	**primer forward** (5′→3′)	**primer reverse** (3′→5′)	**product** (bp)	**amplification protocol** (45 cycles)
JUN-promotor I	5′- AGAGCCTTGTCTCCAGCCGGCCCC - 3′ (Position: -779)	5′- AGAACAGTCCGTCACTTCAC -3′ (Position: -1)	779	denaturation: 45 s, 95°C, primer annealing: 45 s, 58°C, amplification: 180 s, 72°C
JUN promotor II	5′- ACCGTCGCTCCTGAA -3′ (Position: -1736)	5′- GCCACTTGTCTCCGGGT –3′ (Position: -619)	1118	denaturation: 45 s, 95°C, primer annealing: 45 s, 50°C, amplification: 180 s, 72°C
FOS promotor I	5′ - CATATTAGGACATCTGCGTC - 3′ (Position: -468)	5′- CGTGGCGGTTAGGCAAAGCCG -3′ (Position: -1)	468	denaturation: 45 s, 95°C, primer annealing: 45 s, 50°C, amplification: 90 s, 72°C

bp: base pairs; position +1: first base of the start codon **A**TG

Table S7: primer sequences and specific conditions for genotyping

**gene**	**position**	**rs number**	**PCR primer forward** (5′→3′)	**PCR primer reverse** (3′→5′)	**product** (bp)	**amplification protocol** (45 cycles)	**Genotyping Primer**	**genotyping protocol** (44 cycles)
FOS	-135	rs2239615	5′- ACGTTGGATGCTCATTCATAAAACGCTTGTTA- 3′	5′- ACGTTGGATGGGCTCAGTCTTGGCTTC - 3′	115	denaturation: 45 s, 95°C, primer annealing: 45 s, 58°C, amplification: 45 s, 72°C	5′- bioCCGCATCT[L]CAGCGAGCA - 3′	denaturation: 10 s, 94°C, primer annealing: 30 s, 60°C, amplification: 10 s, 72°C
FOS	-60	rs7101	5′- ACGTTGGATGAGCGAACGAGCAGTGAC- 3′	5′- ACGTTGGATGATCATCGTGGCGGTTAG- 3′	124	denaturation: 45 s, 95°C, primer annealing: 45 s, 58°C, amplification: 45 s	5′- bioAGA[L]AGGTGGGCGCTGTG- 3′	denaturation: 10 s, 94°C, primer annealing: 30 s, 60°C, amplification: 10 s, 72°C
JUN	-617 -618	rs4647009	5′- ACGTTGGATGTGCAGCAGCAAAGAACTT- 3′	5′- ACGTTGGATGCAGGAAAGGCTTGCAAA- 3′	103	denaturation: 45 s, 95°C, primer annealing: 45 s, 58°C, amplification: 45 s	5′- bioAGT[L]GGCTCCGGGACTCTG- 3′	denaturation: 10 s, 94°C, primer annealing: 30 s, 60°C, amplification: 10 s, 72°C

bio: biotin, (L): photo cleavable linker. bp: base pair
